# Establishment and Characterization of Humanized Mouse NPC-PDX Model for Testing Immunotherapy

**DOI:** 10.3390/cancers12041025

**Published:** 2020-04-22

**Authors:** Wai Nam Liu, Shin Yie Fong, Wilson Wei Sheng Tan, Sue Yee Tan, Min Liu, Jia Ying Cheng, Sherlly Lim, Lisda Suteja, Edwin Kunxiang Huang, Jerry Kok Yen Chan, Narayanan Gopalakrishna Iyer, Joe Poh Sheng Yeong, Darren Wan-Teck Lim, Qingfeng Chen

**Affiliations:** 1 Institute of Molecular and Cell Biology, Agency for Science, Technology and Research, Singapore 138673, Singapore; wnliu@imcb.a-star.edu.sg (W.N.L.); fongsy@imcb.a-star.edu.sg (S.Y.F.); wstan@imcb.a-star.edu.sg (W.W.S.T.); sytan@imcb.a-star.edu.sg (S.Y.T.); mliu@imcb.a-star.edu.sg (M.L.); chengjy@imcb.a-star.edu.sg (J.Y.C.); sherllyl@imcb.a-star.edu.sg (S.L.); joe.yeong.p.s@sgh.com.sg (J.P.S.Y.); 2 Division of Medical Oncology, National Cancer Centre, Singapore 169610, Singapore; lisda.suteja@nccs.com.sg (L.S.); gopaliyer@singhealth.com.sg (N.G.I.); 3 Department of Reproductive Medicine, KK Women’s and Children’s Hospital, Singapore 229899, Singapore; Edwin.Huang.Kunxiang@kkh.com.sg (E.K.H.); jerrychan@duke-nus.edu.sg (J.K.Y.C.); 4 Experimental Fetal Medicine Group, Yong Loo Lin School of Medicine, National University of Singapore, Singapore 119228, Singapore; 5 Department of Physiology, Yong Loo Lin School of Medicine, National University of Singapore, Singapore 117593, Singapore

**Keywords:** immune checkpoint blockade, nasopharyngeal carcinoma, patient-derived xenografts, humanized mouse model

## Abstract

Immune checkpoint blockade (ICB) monotherapy shows early promise for the treatment of nasopharyngeal carcinoma (NPC) in patients. Nevertheless, limited representative NPC models hamper preclinical studies to evaluate the efficacy of novel ICB and combination regimens. In the present study, we engrafted NPC biopsies in non-obese diabetic-severe combined immunodeficiency interleukin-2 receptor gamma chain-null (NSG) mice and established humanized mouse NPC-patient-derived xenograft (NPC-PDX) model successfully. Epstein–Barr virus was detected in the NPC in both NSG and humanized mice as revealed by Epstein–Barr virus-encoded small RNA (EBER) in situ hybridization (ISH) and immunohistochemical (IHC) staining. In the NPC-bearing humanized mice, the percentage of tumor-infiltrating CD8^+^ cytotoxic T cells was lowered, and the T cells expressed higher levels of various inhibitory receptors, such as programmed cell death protein 1 (PD-1) and cytotoxic T-lymphocyte-associated protein 4 (CTLA-4) than those in blood. The mice were then treated with nivolumab and ipilimumab, and the anti-tumor efficacy of combination immunotherapy was examined. In line with paired clinical data, the NPC-PDX did not respond to the treatment in terms of tumor burden, whilst an immunomodulatory response was elicited in the humanized mice. From our results, human proinflammatory cytokines, such as interferon-gamma (IFN-γ) and interleukin-6 (IL-6) were significantly upregulated in plasma. After treatment, there was a decrease in CD4/CD8 ratio in the NPC-PDX, which also simulated the modulation of intratumoral CD4/CD8 profile from the corresponding donor. In addition, tumor-infiltrating T cells were re-activated and secreted more IFN-γ towards ex vivo stimulation, suggesting that other factors, including soluble mediators and metabolic milieu in tumor microenvironment may counteract the effect of ICB treatment and contribute to the tumor progression in the mice. Taken together, we have established and characterized a novel humanized mouse NPC-PDX model, which plausibly serves as a robust platform to test for the efficacy of immunotherapy and may predict clinical outcomes in NPC patients.

## 1. Introduction

Nasopharyngeal carcinoma (NPC) is characterized by distinct geographical distribution, which is highly associated with chronic infection of Epstein–Barr virus (EBV) [1]. According to the latest statistics, there were around 130,000 newly reported NPC cases globally, with a higher prevalence found in Southern China and South-East Asia [2], and the incidence and mortality rates are higher in males than in females [3]. Some risk factors have been implicated for the NPC in endemic areas, including EBV infection and host genetics [4]. According to the World Health Organization, NPC can be classified into three pathological subtypes, namely non-keratinizing, keratinizing and basaloid squamous cell carcinoma. In those endemic areas, most NPC cases belong to the non-keratinizing subtype that is highly correlated with EBV infection, where the susceptibility to the infection is likely affected by dietary components and smoking [5]. NPC shows EBV type II pattern, where Epstein–Barr nuclear antigen 1 (EBNA1), latent membrane protein 1 (LMP1), LMP2A, LMP2B and Epstein–Barr virus-encoded small RNA (EBER) are expressed, and each of the EBV antigens contribute differentially to the development of NPC and modulation of immunosurveillance [6,7]. Of equal importance, it has been suggested that certain human leukocyte antigens (HLA), such as HLA-A*02:27 or HLA-A*11:01 increase NPC risk [8], while another report summarizes that individuals having HLA-A*02:07, HLA-A*33:03 or HLA-B*38:02 alleles result in higher risk towards NPC [7]. 

NPC is highly sensitive to chemotherapy and the five year-survival rates in Stages I and II NPC exceed 80%, whereas the rate drops to 10% for stage IV disease [9]. Concurrently, radiotherapy is applied to advanced NPC patients alongside with platinum-based chemotherapy, where the latter sensitizes tumors to the toxic effect of radiotherapy [10]. Due to dense infiltration of lymphocytes in tumor stroma and high expression of programmed death ligand 1 (PD-L1) on tumor cells, immune checkpoint blockades (ICB), particularly through programmed cell death protein 1 (PD-1)/PD-L1 axis, are involved for the treatment of NPC patients [11]. In recent years, combination therapies using nivolumab plus ipilimumab, or nivolumab plus chemoradiation have been evaluated in clinical trials [12], which shed light for the possibility of novel combination regimens. Yet, limited EBV-positive models, both in vitro and in vivo, have hampered the progress of NPC research. Over decades, attempts have been made to generate EBV-positive cell lines from NPC biopsies, such as CG1, NPC/HK1 and C666-1 [13,14,15]. It is speculated that the presence of large numbers of lymphoid cells, fibroblasts and connective tissues in the NPC biopsies may lower the successful rate to establish NPC cell lines [13,16]. In addition, the cell lines may lose their EBV episomes upon prolonged passages [17]. In past decades, several patient-derived xenografts (PDX) from primary or metastatic NPC have been established and characterized in athymic nude mice [18,19], while more recently another few EBV-positive PDX have been successfully propagated in non-obese diabetic-severe combined immunodeficiency (NOD *scid*) mice [20]. Nonetheless, these in vivo models provide limited information to immune-oncology research due to the absence of human immune system. Therefore, the development and characterization of humanized mouse cancer models are essential to further research and understanding of NPC biology. For this purpose, CD34^+^ hematopoietic stem cells (HSC) are engrafted into immunocompromised mice by different approaches, followed by inoculation or transplantation of human cancer cell lines or PDX to generate diverse humanized mouse cancer models [21,22]. In the presence of humanized immune system, different immunological characteristics, including circulating and tumor-infiltrating immune cell profile, as well as cytokine profile can be studied in relation to the tumor. Till now, a broad spectrum of cancers, including human gastric carcinoma [23], hepatocellular carcinoma [24], melanoma [25], prostate cancer [26] and renal cell carcinoma [23,27] has been used to generate different humanized mouse cancer models. Nevertheless, humanized mouse NPC model has not yet been established, which hinders preclinical studies to evaluate the efficacy and safety of therapeutic agents for NPC patients. Hence, there is a pressing need to develop and characterize a reliable and robust human-in-mouse NPC platform for screening and testing next-generation combination immunotherapy. 

In the present study, we established and validated a humanized mouse NPC-PDX model, and examined the modulation of the humanized immune system after NPC transplantation. Furthermore, the anti-tumor efficacy of established checkpoint inhibitors nivolumab and ipilimumab was evaluated, and the phenotypes of tumor-infiltrating T cells were compared with paired clinical data.

## 2. Results

### 2.1. Establishment and Characterization of NPC-PDX in Humanized Mice

**Figure 1:**
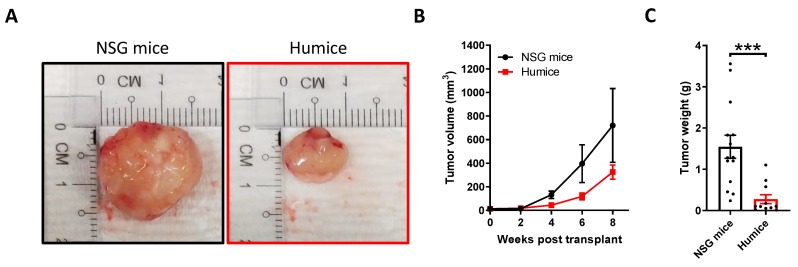

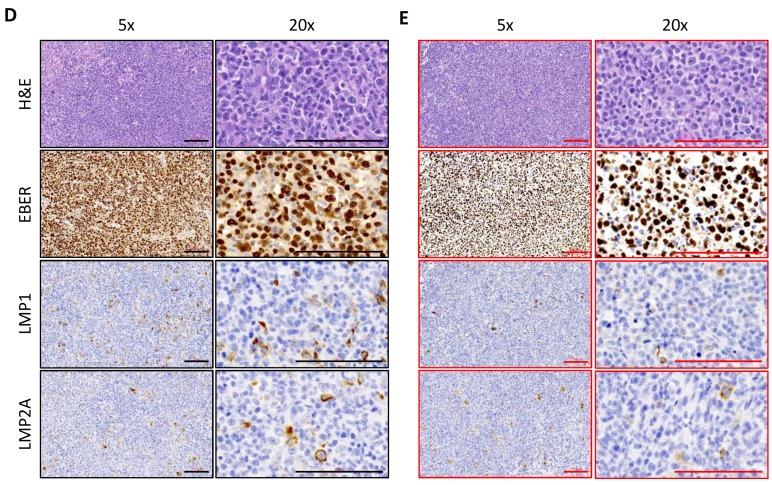
Establishment of humanized mouse nasopharyngeal carcinoma (NPC)-patient-derived xenograft (PDX) model. NPC-PDX were transplanted in non-obese diabetic-severe combined immunodeficiency interleukin-2 receptor gamma chain-null (NSG) mice (*n* = 14) and humanized mice (*n* = 11) subcutaneously. (**A**) Representative images of tumor from NSG mice (Left) and humanized mice (Right) after eight weeks of transplant. The tumor volume (**B**) and tumor weight (**C**) from the mice are shown. *** *p* < 0.001. Representative photomicrographs showing hematoxylin and eosin (H&E), Epstein–Barr virus-encoded small RNA (EBER) in situ hybridization (ISH) and immunohistochemical (IHC) staining on NPC-PDX from the NSG mice (**D**) and humanized mice (**E**). The results of H&E staining confirmed that the PDX belongs to the undifferentiated NPC type, and the presence of Epstein–Barr virus (EBV) was indicated by the expressions of EBER, latent membrane protein (LMP)1 and LMP2A. Bars: 100 μm.

In the present study, we first attempted to engraft NPC-PDX in non-obese diabetic-severe combined immunodeficiency interleukin-2 receptor gamma chain-null (NSG) mice using fresh NPC biopsies obtained from stage IV metastatic and/or recurrent patients. Seven NPC xenografts from a total of 37 biopsies exhibited subcutaneous growth in the NSG mice, with a successful rate of 18.9%. One of the NPC-PDX, propagated from the biopsy, was further transplanted into humanized mice for immunological characterization. Of note, the HLA of the NPC-PDX and immune system in the humanized mice were not identical, but a minimum of two alleles on HLA-A* and HLA-B* were matched [24]. Tumor growth in the humanized mice and NSG counterparts was monitored weekly, and the tumors were harvested eight weeks after transplantation (Figure 1A). From our results, the NPC-PDX grew slower in the humanized mice and the tumor weight was significantly reduced (Figure 1B,C), suggesting that humanized immune system might play a role in modulating the progression of a tumor. We further confirmed that the PDX remained the undifferentiated NPC type (similar to the original tumor) by hematoxylin and eosin (H&E) staining, and the continued presence of EBV was revealed by EBER in situ hybridization (ISH) and immunohistochemical (IHC) staining using LMP1 and LMP2A antibodies across different passages. Remarkably, these EBV proteins were conserved in the tumor from both NSG and humanized mice (Figure 1D,E). 

### 2.2. Activation of the Immune Response in NPC-transplanted Humanized Mice

**Figure 2:**
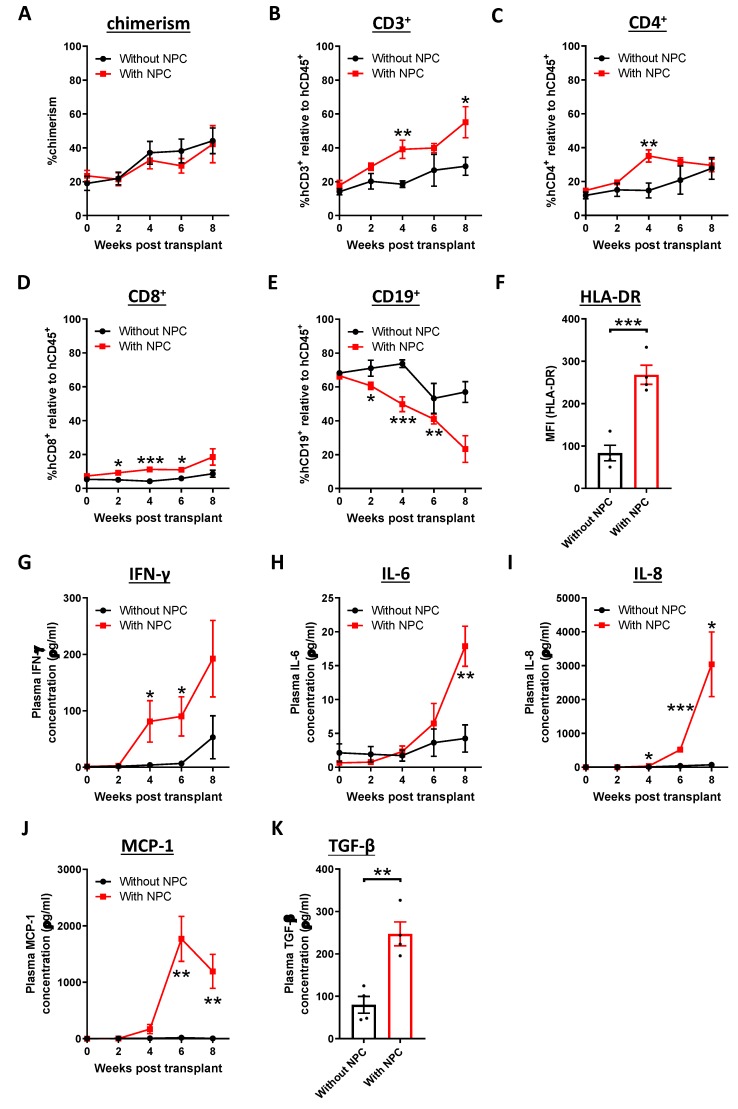
Activation of humanized immune system after NPC transplant. NPC-PDX were transplanted in humanized mice subcutaneously. Blood samples from humanized mice with or without tumor (*n* = 6 from each group) were collected at the indicated weeks post-transplant. The chimerism (**A**), percentages of CD3^+^ T cells (**B**), CD4^+^ T cells (**C**), CD8^+^ T cells (**D**) and CD19^+^ B cells (**E**) were analyzed by flow cytometry. (**F**) The expression of human leukocyte antigens (HLA)-DR on the circulating CD8^+^ T cells from the mice (*n* = 4 from each group) was examined eight weeks after transplant. Cytokines and chemokine levels in plasma, including interferon-gamma (IFN-γ) (**G**), interleukin (IL)-6 (**H**), IL-8 (**I**), monocyte chemoattractant protein-1 (MCP-1) (**J**) and transforming growth factor-beta 1 (TGF-β1) (**K**) were analyzed by LEGENDplex and ELISA (*n* = 4 from each group). Data are expressed as means ± standard error of mean (SEM). * *p* < 0.05; ** *p* < 0.01; *** *p* < 0.001.

To investigate the phenotypic changes of immune cells after NPC engraftment, peripheral blood mononuclear cells (PBMC) from humanized mice were examined by flow cytometric analysis. The gating strategy is shown in Figure S1. In the presence of NPC, there was minimal effect, if any, on the chimerism of the mice (Figure 2A), whilst there was a gradual increase in the percentage of CD3^+^ T cells (Figure 2B). The increase in the CD3^+^ T cells was contributed by both CD4^+^ and CD8^+^ T cells (Figure 2C,D). In contrast, the percentage of CD19^+^ B cells was reduced after NPC transplant (Figure 2E). Other immune cells, including CD14^+^ macrophages, CD56^+^ natural killer (NK) cells and their subsets were also detected in our model (Figure S2A–G). From our results, there were fewer classic macrophages and cytokine-producing NK cells in the NPC-engrafted mice at experimental endpoint. Intriguingly, the CD8^+^ T cells showed an augmented level of HLA-DR expression (Figure 2F) and displayed an effector memory phenotype (Figure S2H), indicating that the humanized immune response was elicited after tumor engraftment. Circulating cytokine and chemokine profile was examined by LEGENDplex and enzyme-linked immunosorbent assay (ELISA), and plasma concentrations of interferon-gamma (IFN-γ), interleukin-6 (IL-6), interleukin-8 (IL-8), monocyte chemoattractant protein-1 (MCP-1) and transforming growth factor-beta 1 (TGF-β1) were upregulated (Figure 2G–K). Spleen was harvested at experimental endpoint and the immune cell profile was investigated. Concordant with the immunomodulation observed in blood, there was an elevation in the percentage of splenic CD3^+^ T cells, accompanied by a reduction in CD19^+^ B cells after tumor transplant, and the increase in the splenic T cells was dominantly contributed by CD8^+^ T cells that exhibited an effector memory phenotype (Figure S3A–E). Moreover, there was a decrease in the percentage of classic and non-classic macrophages, and cytokine-producing NK cells in the NPC-bearing mice (Figure S3F–L). Taken together, our results suggested that the humanized immune system was activated after NPC transplant, as reflected by the increase in the proportion of CD3^+^ T cells and the activation of CD8^+^ T cells in the humanized mice. 

### 2.3. Tumor-Infiltrating T Cells Display Exhausted Phenotypes

**Figure 3:**
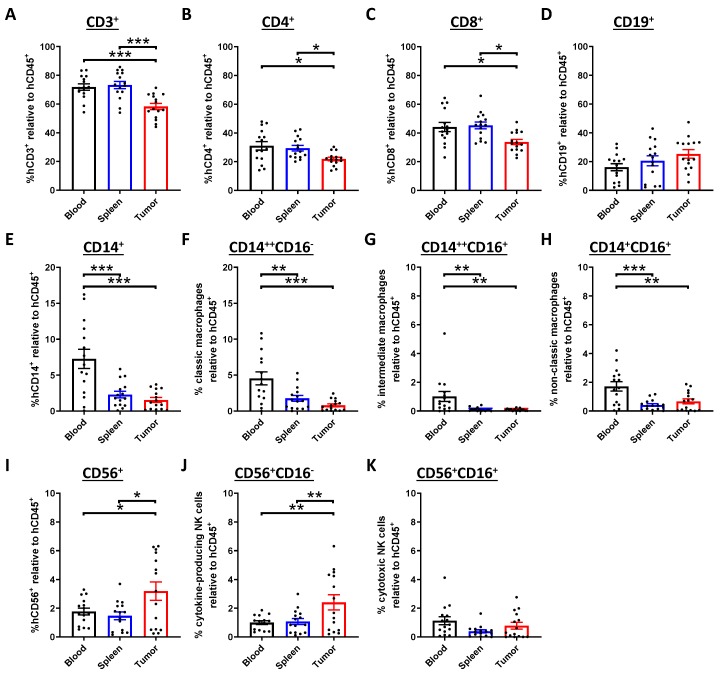
Presence and modulation of human immune cells in tumor. NPC-PDX were transplanted in humanized mice subcutaneously. After eight weeks post-transplant, blood samples, spleens and tumors were collected from the mice (*n* = 15, combined from 3 experiments). The percentages of CD3^+^ T cells (**A**), CD4^+^ T cells (**B**), CD8^+^ T cells (**C**), CD19^+^ B cells (**D**), CD14^+^ macrophages (**E**), classic macrophages (**F**), intermediate macrophages (**G**), non-classic macrophages (**H**), CD56^+^ natural killer (NK) cells (**I**), cytokine-producing NK cells (**J**) and cytotoxic NK cells (**K**) in these organs were examined by flow cytometric analysis. Data are expressed as means ± SEM. * *p* < 0.05; ** *p* < 0.01; *** *p* < 0.001.

**Figure 4:**
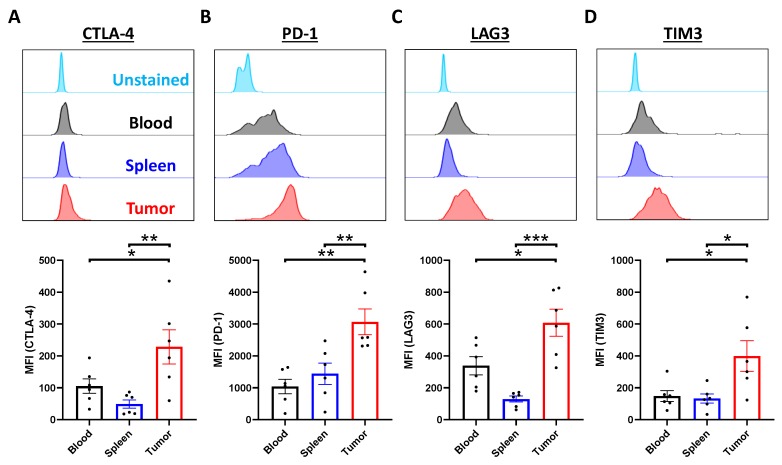
Presence of exhausted CD8^+^ T cells in tumor. NPC-PDX were transplanted in humanized mice subcutaneously. After eight weeks post-transplant, tumors were collected from the mice (*n* = 6). The expression levels of several inhibitory receptors, such as cytotoxic T-lymphocyte-associated protein 4 (CTLA-4) (**A**), programmed cell death protein 1 (PD-1) (**B**), lymphocyte-associated gene 3 (LAG3) (**C**) and T-cell immunoglobulin and mucin domain 3 (TIM3) (**D**) were determined. Data are expressed as means ± SEM. * *p* < 0.05; ** *p* < 0.01; *** *p* < 0.001.

Due to the influence of tumor microenvironment, it is anticipated that the phenotypes and functions of tumor-infiltrating lymphocytes (TIL) are distinguishable from the immune cells in periphery and organs. To characterize the immune profile of TIL in our model, NPC was harvested from humanized mice at the experimental endpoint. In the tumor-bearing humanized mice, the percentages of tumor-infiltrating CD3^+^, CD4^+^ and CD8^+^ T cells were lower than those in blood and spleen (Figure 3A–C), while there were no significant changes in the percentage of CD19^+^ B cells (Figure 3D). In addition, the percentages of various CD14^+^ macrophage subsets in the tumor were lowered (Figure 3E–H), whereas the cytokine-producing NK cells, but not the cytotoxic NK cells, were increased statistically (Figure 3I–K). Similarly, the percentages of the tumor-infiltrating T cells were also reduced, as demonstrated using another NPC-PDX (Figure S4A–K). Consistent with the primary tumor, T cells were also the major immune cell subset in the NPC-PDX, albeit the tumor-infiltrating macrophages and NK cells accounted for around 5% in our model [28,29]. Hence, the expressions of immune checkpoint receptors on the cytotoxic T lymphocytes were further examined. By flow cytometric analysis, it was found that the tumor-infiltrating CD8^+^ T cells from both PDX expressed higher levels of cytotoxic T-lymphocyte-associated protein 4 (CTLA-4), PD-1, lymphocyte-associated gene 3 (LAG3) and T-cell immunoglobulin and mucin domain 3 (TIM3) when compared to those in the circulation and spleen (Figure 4A–D; Figure S4L–O), revealing that the T cells exhibited exhausted phenotype in the immunosuppressive tumor milieu [30]. 

### 2.4. Examination of Anti-Tumor Efficacy Using ICB in Humanized Mouse NPC-PDX Model

In recent years, ICB, with monoclonal antibodies such as pembrolizumab, nivolumab and camrelizumab have been used to treat advanced stage NPC patients in multiple clinical trials [31,32,33]. In our humanized mouse NPC-PDX model, the tumor-infiltrating CD8^+^ T cells expressed higher levels of PD-1 and CTLA-4, as well as LAG3, and these exhausted phenotypes might hinder the anti-tumor efficacy of ICB by suppressing their cytotoxicity and cytokine-producing capability. Hence, it is of great interest to examine whether ICB are able to restore the functions of the tumor-infiltrating T cells and reduce tumor burden in humanized mice. These humanized mice were transplanted with the NPC-PDX and upon formation of visible tumors, which takes usually 2 to 3 weeks after transplantation, the mice were then injected with a cocktail of nivolumab (anti-PD-1 antibody; 3 mg/kg) and ipilimumab (anti-CTLA-4 antibody; 1 mg/kg) on a weekly basis. Four weeks after treatment, the tumors were harvested for downstream analysis (Figure S5A). From our results, the NPC-PDX did not respond to the ICB treatment in terms of the tumor weight (Figure S5B,C).

### 2.5. Modulation of Immune Cell and Cytokine Profile after Treatment

**Figure 5:**
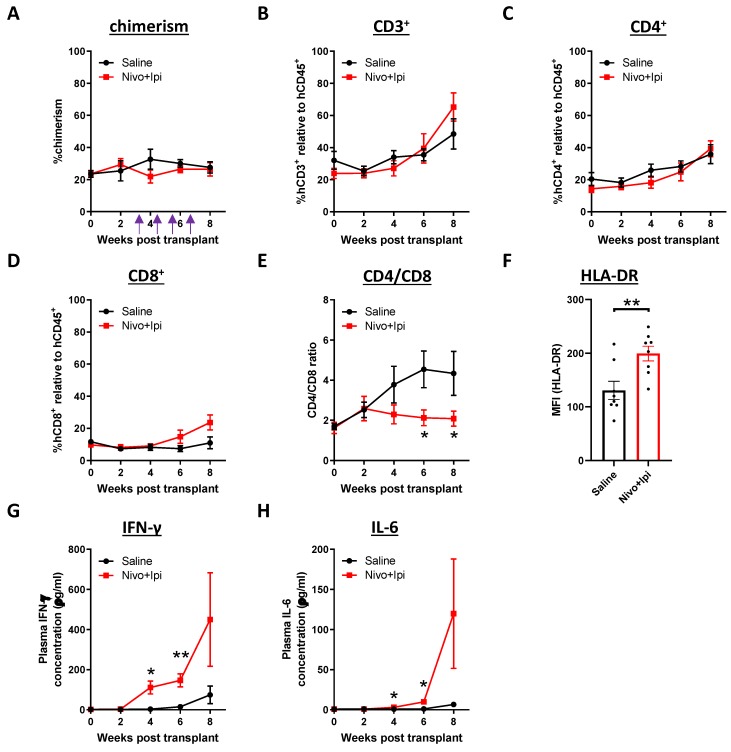
Modulation of humanized immune system in blood after combination immunotherapy. NPC-transplanted humanized mice were injected with saline or nivolumab plus ipilimumab. Purple arrows indicate the injection time point. Blood samples (*n* = 8 from each group) were collected at the indicated weeks post-transplant. The chimerism (**A**), percentages of CD3^+^ T cells (**B**), CD4^+^ T cells (**C**), CD8^+^ T cells (**D**), CD4/CD8 ratio (**E**) and the expression of HLA-DR on the CD8^+^ T cells at the experimental endpoint (**F**) were analyzed. Cytokines levels in plasma, including IFN-γ (**G**) and IL-6 (**H**) were determined (*n* = 4 from each group). Data are expressed as means ± SEM. * *p* < 0.05; ** *p* < 0.01.

Next, we evaluated the modulation of immune responses in the NPC-bearing humanized mice after combination therapy. In the ICB-treated mice, there were no significant changes in the chimerism (Figure 5A). Intriguingly, there was an increase in the percentage of CD3^+^ and CD8^+^ T cells observed at later time point, resulting in a decrease in CD4/CD8 ratio (Figure 5B–E). In addition, the expression level of HLA-DR on the CD8^+^ T cells was up-regulated (Figure 5F) and immediate increases in the plasma IFN-γ and IL-6 were found (Figure 5G,H), which indicated that the immune system was further activated upon the treatment.

### 2.6. Presence of Re-Activated Tumor-Infiltrating T Cells after Treatment

**Figure 6:**
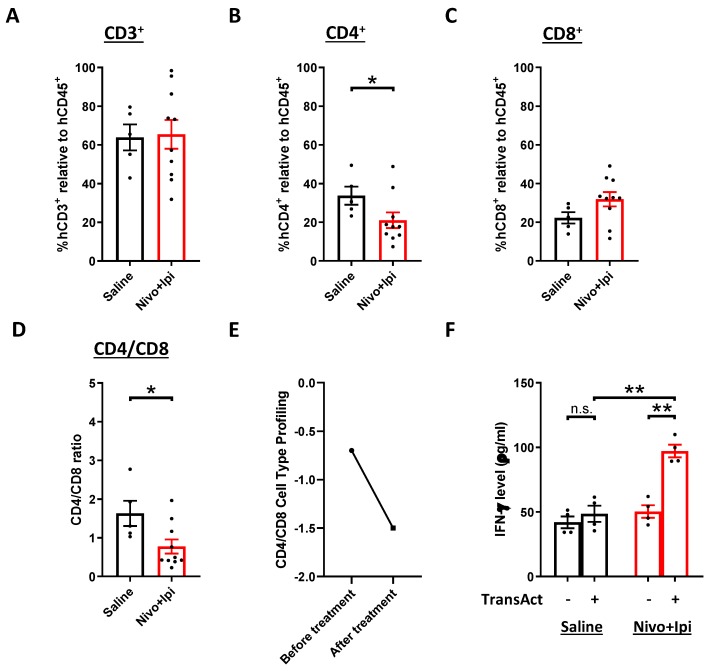
Modulation of immune cell profile in tumor after combination immunotherapy. Tumors were harvested from saline-treated humanized mice (*n* = 5) and nivolumab plus ipilimumab-treated humanized mice (*n* = 10; combined from 2 experiments) at the experimental endpoint. The percentages of CD3^+^ T cells (**A**), CD4^+^ T cells (**B**), CD8^+^ cells T cells (**C**) and CD4/CD8 ratio (**D**) were analyzed. (**E**) The CD4/CD8 cell type profiling from corresponding donor was revealed by Nanostring analysis. (**F**) Tumor-infiltrating CD3^+^ T cells were isolated from the saline-treated group (*n* = 4) and nivolumab plus ipilimumab-treated group (*n* = 4). The release of IFN-γ after TransAct stimulation was determined by ELISA. Data are expressed as means ± SEM. *n.s.* means non-significant; * *p* < 0.05; ** *p* < 0.01.

Apart from PBMC, the immune cell profile in tumor was analyzed after ICB treatment. From our results, there were no significant changes in the percentage of CD3^+^ T cells (Figure 6A). However, the CD4/CD8 ratio in the NPC-PDX was decreased (Figure 6B–D). Interestingly, paired tumor biopsies from the patient donor showed a similar reduction in the CD4/CD8 ratio after dual PD-1/CTLA4 blockade (Figure 6E). Similar trend of the CD4/CD8 ratio was also seen in subsequent NPC-PDX derived from other patient donor treated with dual ICB (Figure S6), which further supports that the humanized mice can simulate the immune responses to ICB. Last but not least, tumor-infiltrating CD3^+^ T cells were isolated for ex vivo activation. It was found that the T cells from the ICB-treated humanized mice were capable of secreting more IFN-γ after stimulation (Figure 6F), which suggests that the tumor-infiltrating T cells were re-activated and the ICB might restore, at least partially, the function of the T cells. 

## 3. Discussion

EBV-associated NPC exhibits a distinct geographical distribution and most cases occur in Southern China and South-East Asia. Nevertheless, due to limited in vitro and in vivo models, the progress of basic and translational research on NPC has been limited. Reliable EBV-positive patient-derived NPC models for both oncology and immunology studies, would accelerate the evaluation of novel immunotherapy regimens and further development of effective treatments. 

Previous attempts to establish NPC-PDX in vivo have been successful in nude mice and NOD *scid* mice. In the present study, fresh NPC biopsies obtained from stage IV metastatic and/or recurrent patients were transplanted in NSG mice and the tumor take rate for PDX engraftment was 18.9%, which is comparable to those aforementioned studies [18,19,20,34]. Yet, the lack of comprehensive immune system in immunocompromised mice hinders the understanding of immunological responses during cancer progression, as well as the development and evaluation of the efficacy of cancer immunotherapies [21,35]. In the presence of humanized immune system, the growth of the tumor was significantly inhibited. Notably, it has been reported that the NPC-PDX may lose their EBV episomes after prolonged passages [17], potentially altering the tumor-immune milieu and limiting the interpretation of experiments done with these models. To confirm the presence of EBV in the tumor from our PDX, EBER ISH and IHC staining for LMP1 and LMP2A were performed prior to subsequent experiments. Our results showed that the EBV were retained in the tumors in both NSG and humanized mice upon serial passages, and the NPC-PDX was EBV-positive at the time of experiments.

The role of the human immune system in shaping immunogenicity of tumor has been extensively reviewed, where cancer immunoediting can be categorized into elimination, equilibrium and escape phases through activation of innate and adaptive immune responses [36,37]. From our results, there was an increase in human CD8^+^HLA-DR^+^ T cells in the NPC-bearing humanized mice, compared to their non-engrafted counterparts, suggesting that the immune system was activated. In addition, the transplantation of NPC led to an elevation of numerous cytokines, including IFN-γ, IL-6 and IL-8, as well as MCP-1, a chemokine that regulates migration and infiltration of monocytes/macrophages [38]. IFN-γ was shown to protect the host against the growth of transplanted tumors, chemically-induced tumors and spontaneous tumors, which forms the basis of tumor immunosurveillance and promotes elimination of tumor cells at early stage of engraftment [39,40]. The increase in circulating IFN-γ level in humanized mice may account for the reduction in tumor burden when compared to the immunocompromised NSG mice. Remarkably, more IL-6 and IL-8 were secreted in the NPC-PDX mice at later time point. These cytokines are believed to promote tumor growth by supporting angiogenesis, although the pro-tumorigenic role of IL-6 is still inconclusive as reported elsewhere [41,42]. TGF-β plays a dual role in modulating cancer development [43]. At the experimental endpoint, it was found that the plasma concentration of total TGF-β1 was higher in the NPC-bearing humanized mice, which is in line with the data in clinical settings [44].

Previous clinical studies have demonstrated that NPC is densely infiltrated by T cells [45], where the presence of more TIL, particularly CD3^+^ T cells, was associated with favorable outcomes [46,47]. In our model, TIL was detected in the humanized mice, and the percentage of intratumoral T cells was lower than those in circulation and spleen. More importantly, the expressions of several inhibitory receptors on the intratumoral CD8^+^ T cells, including PD-1, CTLA-4, LAG3 and TIM3 were upregulated, resulting in exhausted phenotypes that are frequently observed in cancer patients [48,49]. PD-1 is one of the major inhibitory receptors that regulates T-cell exhaustion and the T cells with high PD-1 expression fail to eradicate cancer [48], thus making PD-1 an attractive target in cancer immunotherapy using anti-PD-1 monoclonal antibody, although the reported objective response rates were around 20% to 30% in different single-arm trials [4]. Similarly, CTLA-4 plays a critical role in attenuating the early activation of naïve and memory T cells [50] and NPC patients with higher tumor CTLA-4 expression had poorer prognosis [51]. To evaluate the anti-tumor efficacy of combination therapy and align with our clinical trial, the NPC-PDX humanized mice were administrated with nivolumab plus ipilimumab. The tumors in humanized mice did not respond to the combination therapy in terms of the tumor burden, which is consistent with the lack of response in the donor patient in clinic. In addition, they displayed a similar trend in the modulation of CD4/CD8 ratio after treatment, suggesting that humanized mice could be used to mimic some of the responses in patients after treatment. Interestingly, ex vivo T-cell activation assay revealed that tumor-infiltrating CD3^+^ T cells isolated from the ICB-treated group were more responsive to the stimulation and secreted more IFN-γ. Yet, the re-activation of T cells may remain insufficient to exert significant anti-tumor effect in vivo, suggesting alternate pathways of resistance including other inhibitory checkpoints, such as LAG3 and TIM3. Of note, LAG3 was upregulated in our NPC-PDX model, and LAG3 upregulation was also seen in a recent trial of spartalizumab in NPC [52]. Indeed, relatlimab, an anti-LAG3 antibody, is currently under clinical development in several tumors [53]. Apart from the inhibitory ligands, it is also anticipated that other immunosuppressive signals in tumor microenvironment, such as soluble mediators and metabolic milieu may contribute to the dysfunction states of the intratumoral T cells [30], which may be further investigated in the humanized mouse NPC-PDX model. 

**Figure 7:**
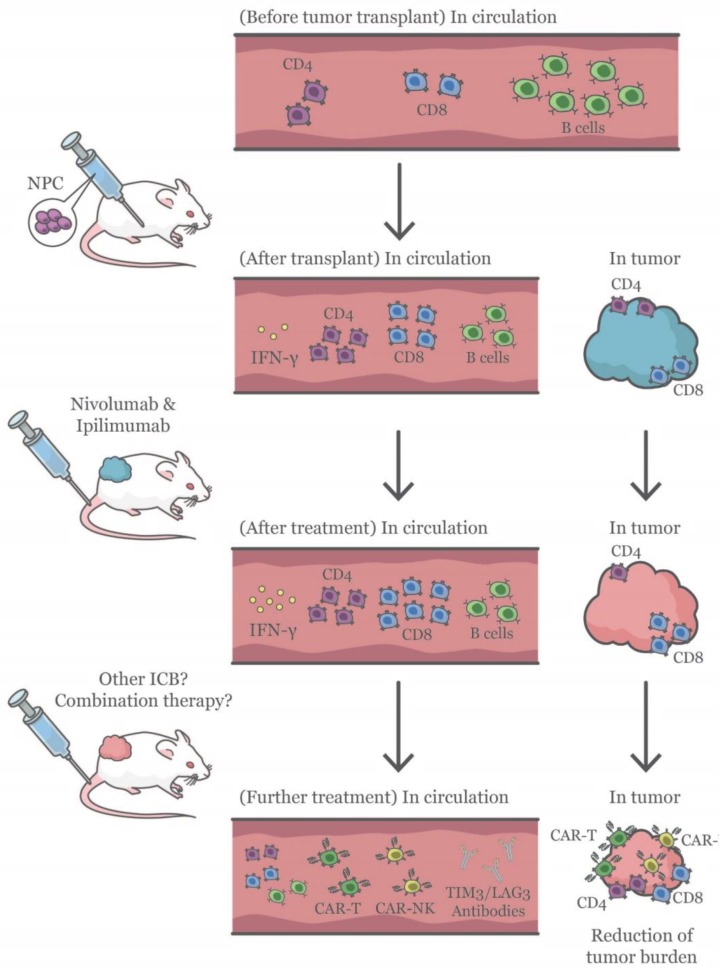
Schematic representation of the immunomodulation in humanized mice after NPC transplantation and immune checkpoint blockade (ICB) treatment. Ten to twelve weeks after HSC injection, major human immune cell subsets were repopulated in the circulation of humanized mice. Subsequently, NPC-PDX was transplanted in one flank of the humanized mice. The humanized immune system was activated as revealed by an increase in plasma IFN-γ. In addition, more CD4^+^ and CD8^+^ T cells were present in the circulation, accompanied by a decrease in the percentage of CD19^+^ B cells after the transplant. At experimental endpoint, it was found that less CD4^+^ and CD8^+^ T cells were present in the tumor (Blue in color) than those in the circulation. Combination therapy using nivolumab and ipilimumab further activated the immune system in NPC-bearing humanized mice, with a higher level of plasma IFN-γ. Moreover, a reduction of CD4/CD8 ratio was observed in the tumor (Red in color) after ICB treatment. In additional to the current combination immunotherapy, other ICB, such as anti-LAG3 and TIM3 antibodies, or cell-mediated therapy involving chimeric antigen receptor (CAR)-engineered T cells and NK cells can be evaluated using the humanized mouse NPC-PDX model.

To the best of our knowledge, this is the first report to describe the establishment and characterization of a humanized mouse NPC-PDX model (Figure 7). However, several limitations remain. Firstly, the take rate can be improved. Hsu et al. reported that PDX engraftment-positive patients had shorter survival than PDX engraftment-negative patients, regardless of their EBV DNA load and previous treatments that the patients received [34]. Hence, it would be of interest to unravel the underlying factors that govern the tumor take rate, which can plausibly increase the bioavailability of NPC-PDX for EBV and NPC research. While most human hematopoietic lineages are engrafted, not all are fully developed in the humanized mice. Due to the absence of full HLA matching and human primary lymphoid organs, human pre-T cells are educated in mice thymus and rely on major histocompatibility complex (MHC) for antigen presentation. This results in limited education of T cells in the mice [54], while some publications demonstrated that human T cells in humanized mice could generate human HLA-restricted responses [55,56]. Bone marrow (BM) microenvironment is capable of supporting T-cell development in the absence of thymus, however, the difference in human and mouse BM microenvironment may further hinder T-cell education and function [57]. Notably, the lack of species-specific growth factors, cytokines and chemokines also hampers the maturation of certain immune cell subsets, including myeloid cells and NK cells [58]. To overcome these hurdles would involve manipulating the human-mouse immune interface. HLA class I and II transgenes have been introduced into NSG mice, which possibly allow the generation of functional HLA-restricted T cells [59]. In addition, efficient methods to improve mouse environment to support better human cell functions have been developed, including hydrodynamic delivery of constructs expressing human-specific cytokines [60,61], and multiple strains of transgenic mice bearing knock-in genes, which constitutively increase the levels of human cytokines in the mice and facilitate the growth of specific immune cell subsets are being developed [62]. These improved models may better recapitulate the human immune responses during cancer progression [63,64]. HLA mismatch could be overcome with PBMC or induced pluripotent stem cells (iPSC) that can be obtained or derived autologously to generate the humanized mouse NPC-PDX model with fully matched HLA. Nevertheless, injection of PBMC results in graft-versus-host-disease [54], whilst the repopulating capability of iPSC in mice is limited [65]. These barriers have to be overcome before a fully HLA-matched humanized mouse cancer model can be established. 

## 4. Materials and Methods

### 4.1. Cord Blood Processing and Isolation of Human CD34^+^ HSC

Human umbilical cord blood was collected from KK Women’s and Children’s Hospital (KKH), Singapore, with written consent obtained from guardians of donors and in strict accordance with the institutional ethical guidelines of KKH. SingHealth and National Health Care Group Research Ethics Committees Singapore specifically approved this study (IRB no: 2013/778/D). The cord blood was purified by density centrifugation with Lymphoprep (Stemcell Technologies, Vancouver, BC, Canada) and red blood cells (RBC) were lysed by EasySep RBC lysis buffer (Stemcell Technologies). Thereafter, human CD34^+^ HSCs were enriched using EasySep Human Cord Blood CD34 Positive Selection kit (Stemcell Technologies) according to manufacturer’s instructions. 

### 4.2. Mice and Transplantation of CD34^+^ Cells 

NSG mice were purchased from The Jackson Laboratory and bred in a specific pathogen-free condition at Biological Resource Centre (BRC) in Agency for Science, Technology and Research (A*STAR), Singapore. To generate humanized mice, one- to three-days-old NSG pups were sub-lethally irradiated at 1 Gy and human CD34^+^ HSC (2 × 10^5^/mouse) were inoculated into the pups by intra-hepatic injection [66]. Human immune cells engraftment (chimerism) in the mice was determined ten to twelve weeks after injection. In essence, blood samples were collected and composition of immune cells was analyzed by flow cytometry. The chimerism was calculated by (% human CD45^+^/(% human CD45^+^ + % mouse CD45^+^)), and humanized mice with the chimerism between 20% and 50% were used for subsequent experiments. 

All animal experiments were conducted in strict accordance to the guidelines on Care and Use of Animals for Scientific Purposes, which are released by National Advisory Committee on Laboratory Animal Research, Agri-Food and Veterinary Authority of Singapore and International Animal Care and Use Committee (IACUC) at A*STAR. The IACUC specifically approved this study (IACUC no: 181367 and 191440). 

### 4.3. Transplantation of NPC-PDX and Drug Treatment

NPC samples were collected from National Cancer Center Singapore (NCCS, Singapore), with written consent obtained from patients and in strict accordance with the institutional ethical guidelines of NCCS. SingHealth and National Health Care Group Research Ethics Committees Singapore specifically approved this study (IRB no: 2016/2887). Fresh NPC sample was cut into fragments (3 × 3 mm) and transplanted in one flank of NSG mouse subcutaneously using aseptic technique. Size and volume of the tumor were recorded weekly using a caliper and passaged in the flank of recipient NSG mice once the tumor reached 10 × 10 mm in size. Tumor volume was calculated by using the formula: Tumor volume = (length × width^2^)/2, where length represents the longest tumor diameter and width represents the perpendicular tumor diameter [67].

To evaluate the anti-tumor efficacy of immune checkpoint blockades, NPC was transplanted in one flank of humanized mice subcutaneously. Once the tumor reached 3 × 3 mm in size, nivolumab (anti-PD-1 antibody; 3 mg/kg) and ipilimumab (anti-CTLA-4 antibody; 1 mg/kg) were administered into the mice intravenously weekly for four consecutive weeks. 

### 4.4. H&E, EBER ISH and IHC Staining

Tumors from NSG and humanized mice were harvested at experimental endpoint, which were formalin-fixed (Sigma, Saint Louis, MO, USA) at room temperature for 24 h. For H&E staining, the fixed tissues were paraffin-embedded (Leica, Germany), sliced into 5 µm sections (Leica) and stained with hematoxylin and eosin (Thermo Fisher Scientific, Waltham, MA, USA). The EBER ISH was conducted for the detection of EBV-positive cells, using Leica Bond-Max autostainer as previously described [68]. For the IHC staining, the tumor sections were stained for LMP1 and LMP2A using their respective antibodies purchased from Abcam (Cambridge, MA, USA), followed by hematoxylin counterstain. All the stained images were captured using a ZEN fluorescence microscope (Zeiss, Germany) with ZEN 2 acquisition software (Zen Blue Version, Zeiss, Germany). 

### 4.5. Isolation of Mononuclear Cells from Blood, Spleen and Tumor 

Humanized mice were cheek-bleed at specified time point. Blood samples were collected in EDTA tubes (Greiner Bio-One, Monroe, NC, USA) and centrifuged at 10, 000 × g for 5 min. The plasma was stored at −80 °C for subsequent cytokines quantification. The cell pellet was resuspended in ACK lysis buffer (Gibco, Grand Island, NY, USA) prior to DAPI and surface marker staining. 

At experimental endpoint, the spleen and tumor were harvested from the mice, and mononuclear cells (MNC) were isolated as reported elsewhere [69]. For the isolation of spleen MNC, the spleen was meshed with a syringe tip and the cell suspension was filtered through a 70 µm cell strainer (Thermo Fisher Scientific). To isolate TIL, the tumor was cut into 2 × 2 mm fragments and digested using Tumor Dissociation Kit, human (Miltenyi Biotec, Germany) and gentleMACS Dissociator (Miltenyi Biotec). The cell suspension was filtered through a 70 µm cell strainer and the TIL was enriched by density centrifugation with Percoll (GE Healthcare, Piscataway, NJ, USA). All the MNC were subjected to RBC lysis using ACK lysis buffer prior to DAPI and surface marker staining.

### 4.6. Flow Cytometry

Before surface marker staining, dead MNC were labeled with the dye provided in LIVE/DEAD Fixable Blue Dead Cell Stain kit (Sigma) at room temperature for 15 min. The MNC were then washed once with fluorescence-activated cell sorting (FACS) buffer and stained with antibodies at 4 °C for 15 min. The antibodies used for FACS are listed in Table S1. Flow cytometric analysis was performed on a LSRII flow cytometer using FACSDiva software (Becton Dickinson, Sparks, MD, USA). A total of 100,000 events were collected per sample and analyzed using Flowjo software version 10 (Treestar, Ashland, OR, USA).

### 4.7. Cytokines and Chemokines Quantification

Cytokines and chemokines levels in the plasma from humanized mice were determined by LEGENDplex Human Inflammation Panel (13-plex) according to manufacturer’s instructions from BioLegend (San Diego, CA, USA). The profile was analyzed by flow cytometry and the LEGENDplex data analysis software (BioLegend).

### 4.8. Nanostring Analysis

RNA was extracted from frozen tumor biopsy using AllPrep DNA/RNA Mini Kit (Qiagen, Valencia, CA, USA) according to the manufacturer’s protocol. In brief, a total of 125 ng RNA (>300 bp) was assayed using nCounter PanCancer IO360 on the nCounter MAX Analysis System (NanoString Technologies, Inc., Seattle, WA, USA). The data was normalized and processed using Advance Analysis module in nSolver Software version 4.0 (NanoString Technologies, Inc.) [70]. Cell type profiling score was calculated as previously described [71]. 

### 4.9. ELISA for Human TGF-β1 and IFN-γ 

Total TGF-β1 level in the plasma from humanized mice was quantified by TGF beta 1 Human ELISA Kit (Abcam) according to manufacturer’s instructions. 

Tumor-infiltrating T cells in humanized mice were isolated using EasySep Human T Cell Isolation kit (Stemcell Technologies). Purified T cells (5 × 10^5^ cells/mL) were seeded into a 96-well plate and stimulated with T Cell TransAct (Miltenyi Biotec) for 24 h. After incubation, supernatant was collected and centrifuged once to remove cell debris. Human IFN-γ level in the cell-free supernatant was determined by LEGEND MAX Human IFN-γ ELISA Kit (BioLegend).

### 4.10. Statistical Analysis

All statistical analysis was performed using GraphPad Prism version 8 (GraphPad, Inc., San Diego, CA, USA) and data are expressed as mean ± SEM. Unpaired Student’s *t*-test and one-way analysis of variance (ANOVA) with *post hoc* Tukey’s Multiple Comparison Test were used, whenever appropriate. The differences are considered as statistically significant at *p* < 0.05.

## 5. Conclusions

We have successfully established and characterized a novel humanized mouse NPC-PDX model where the anti-tumor efficacy of combination cancer therapy can be robustly examined. It is anticipated that other therapeutic options, including but not limiting to cell-mediated therapy or other immune checkpoint blockades can be evaluated in our model.

## Supplementary Materials

The following are available online at https://www.mdpi.com/2072-6694/12/4/1025/s1, Figure S1: Gating strategy for NK cell, macrophage and T cell subsets, Figure S2: Modulation of immune cell subsets in blood in the presence of NPC in humanized mice, Figure S3: Modulation of immune cell subsets in spleen in the presence of NPC in humanized mice, Figure S4: Modulation of tumor-infiltrating immune cells in another NPC-PDX, Figure S5: Evaluation of the anti-tumor efficacy of nivolumab and ipilimumab in humanized mice, Figure S6: Modulation of immune cell profile in tumor after combination immunotherapy using another NPC-PDX, Table S1: FACS antibodies used in the present study.
