# Supplementary material for: Establishment and Characterization of Humanized Mouse NPC-PDX Model for Testing Immunotherapy
Source: Cancers (Basel). 2020 Apr 22;12(4):1025. doi: 10.3390/cancers12041025 (PMC7225949; doi:10.3390/cancers12041025)
Supplement: Supplementary file 1 [file cancers-12-01025-s001.pdf]

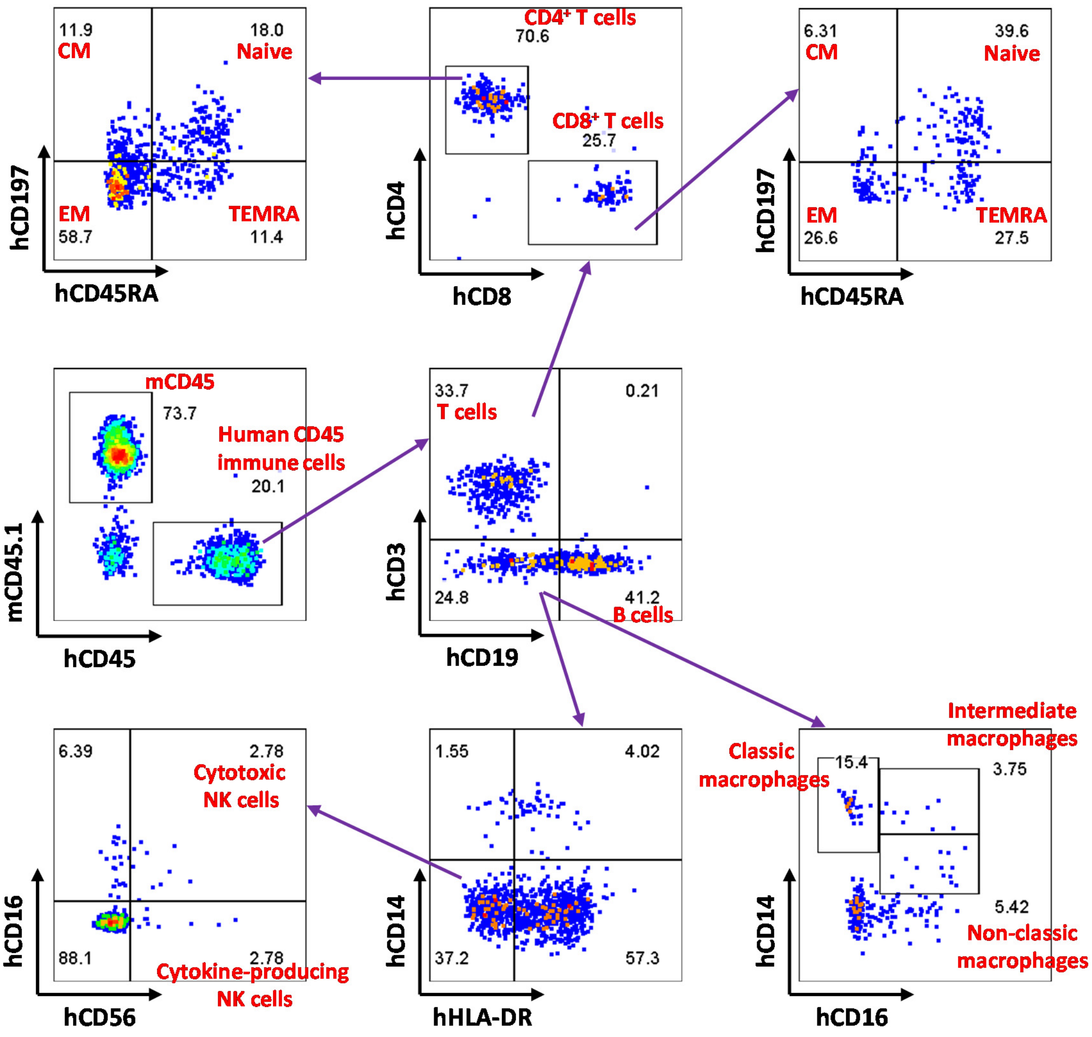

Figure S1. Gating strategy for NK cell, macrophage and T cell subsets.

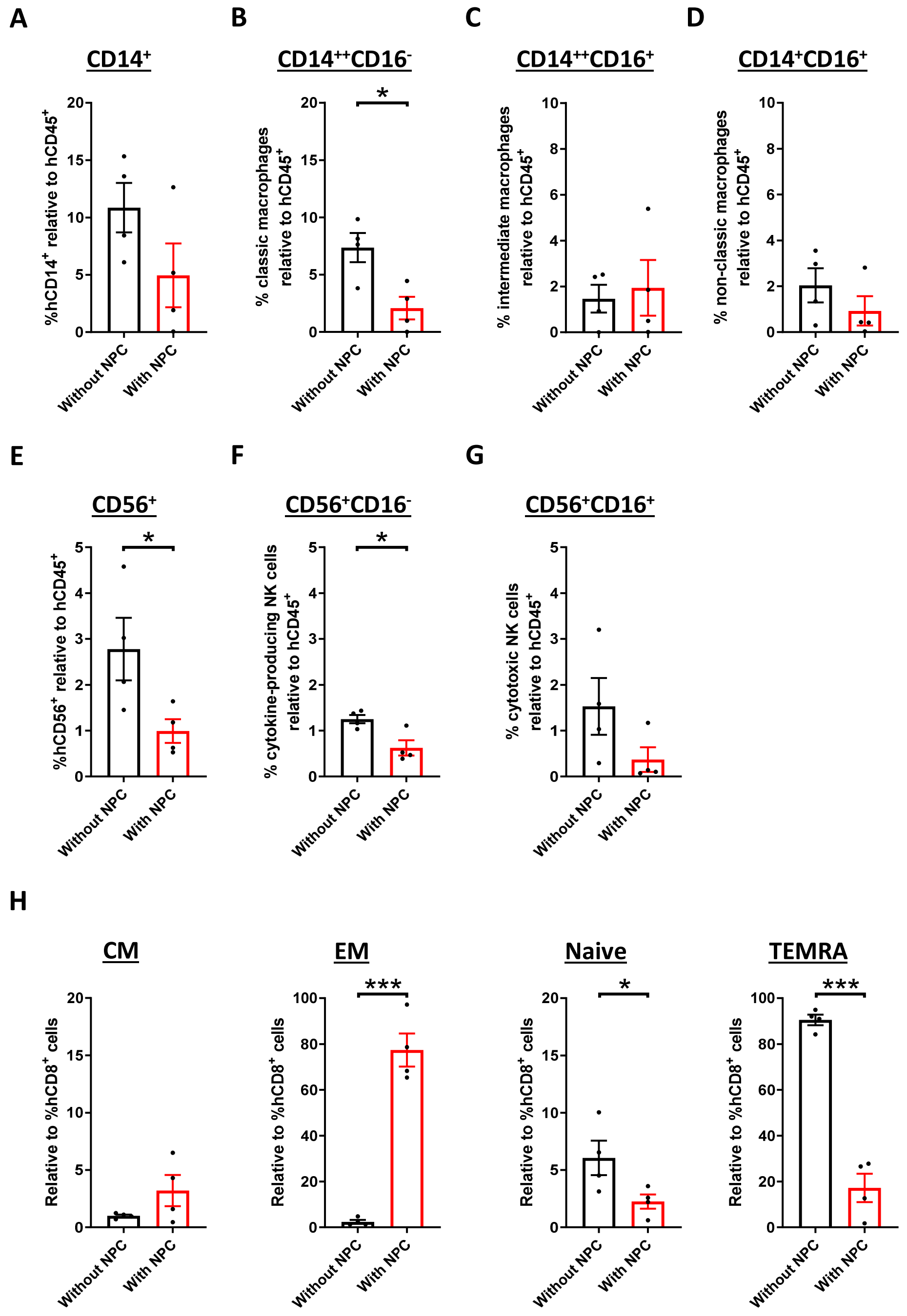

Figure S2. Modulation of immune cell subsets in blood in the presence of NPC in humanized mice. NPC-PDX were transplanted in humanized mice subcutaneously. Blood samples from humanized mice with or without tumor ($n = 4$ from each group) were collected at the indicated weeks post-transplant. The percentage of $CD14^+$ macrophages (**A**), classic macrophages (**B**), intermediate macrophages (**C**), non-classic macrophages (**D**), $CD56^+$ NK cells (**E**), cytokine-producing NK cells (**F**), cytotoxic NK cells (**G**) and the subsets of the $CD8^+$ T cells (**H**) was analyzed by flow cytometry. Data are expressed as means ± SEM. * $p < 0.05$; *** $p < 0.001$.

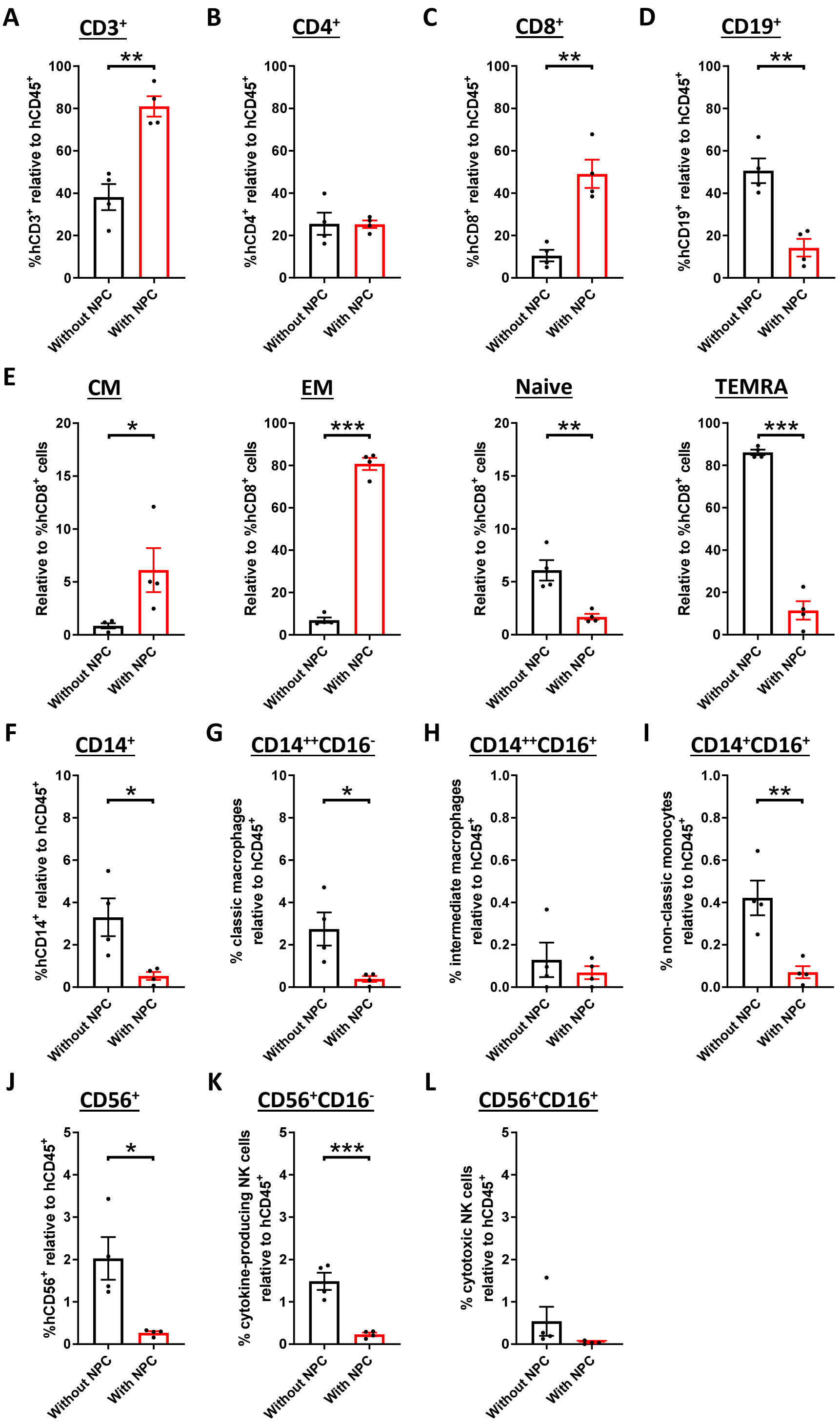

Figure S3. Modulation of immune cell subsets in spleen in the presence of NPC in humanized mice. NPC-PDX were transplanted in humanized mice subcutaneously. After eight weeks post-transplant, spleen was harvested from the mice with or without tumor (*n* = 4 from each group). The percentage of $CD3^+$ T cells (**A**), $CD4^+$ T cells (**B**), $CD8^+$ T cells (**C**), $CD19^+$ B cells (**D**), the subsets of the $CD8^+$ T cells (**E**), $CD14^+$ macrophages (**F**), classic macrophages (**G**), intermediate macrophages (**H**), non-classic macrophages (**I**), $CD56^+$ NK cells (**J**), cytokine-producing NK cells (**K**) and cytotoxic NK cells (**L**) was analyzed by flow cytometry. Data are expressed as means ± SEM. * $p < 0.05$; ** $p < 0.01$; *** $p < 0.001$.

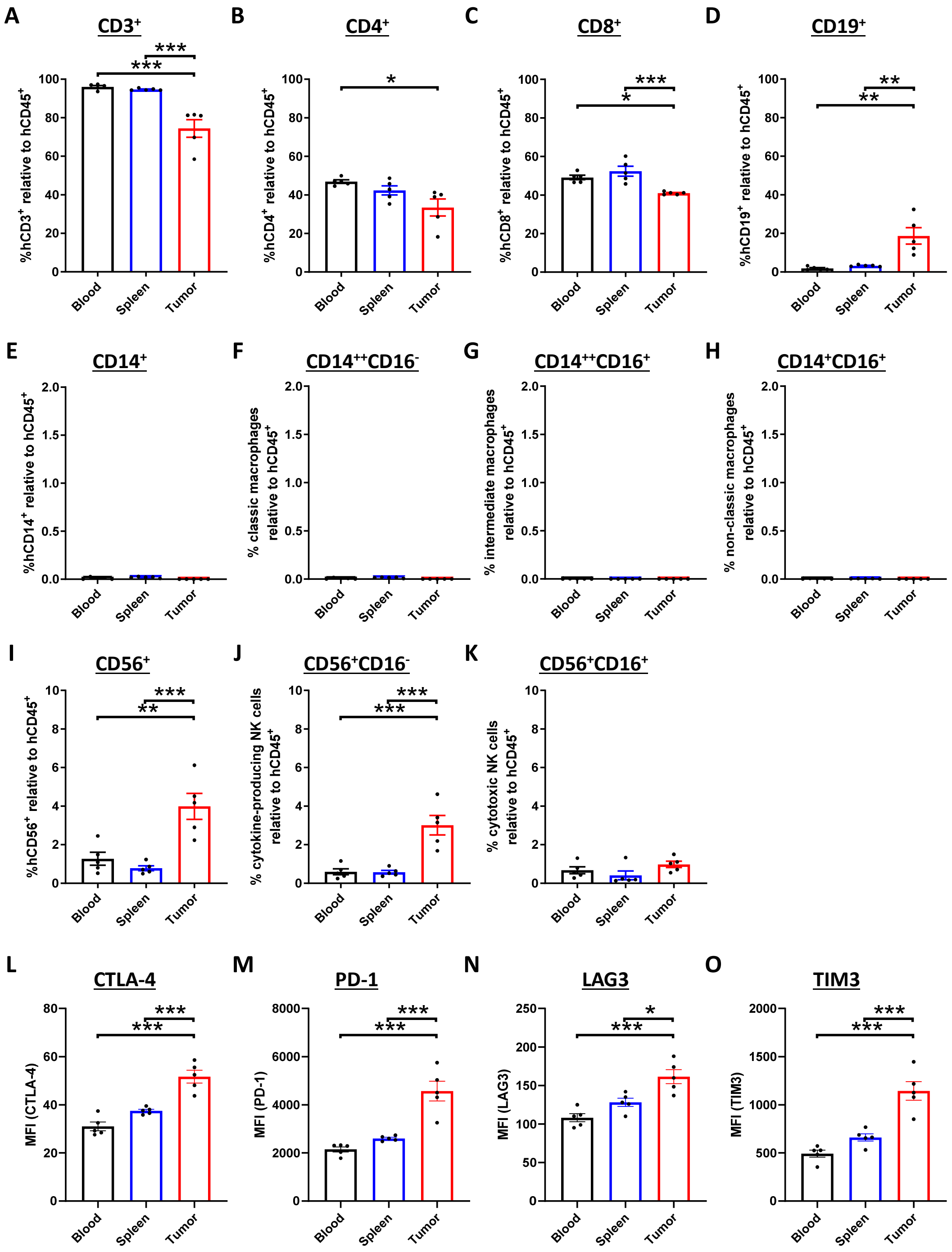

Figure S4. Modulation of tumor-infiltrating immune cells in another NPC-PDX. NPC-PDX were transplanted in humanized mice subcutaneously. After eight weeks post-transplant, blood samples, spleens and tumors were collected from the mice (n = 5). The percentages of CD3$^+$ T cells (**A**), CD4$^+$ T cells (**B**), CD8$^+$ T cells (**C**), CD19$^+$ B cells (**D**), CD14$^+$ macrophages (**E**), classic macrophages (**F**), intermediate macrophages (**G**), non-classic macrophages (**H**), CD56$^+$ NK cells (**I**), cytokine-producing NK cells (**J**) and cytotoxic NK cells (**K**) in these organs were examined by flow cytometric analysis. The expression levels of several inhibitory receptors, such as CTLA-4 (**L**), PD-1 (**M**), LAG3 (**N**) and TIM3 (**O**) were determined. Data are expressed as means ± SEM. * $p < 0.05$; ** $p < 0.01$; *** $p < 0.001$.

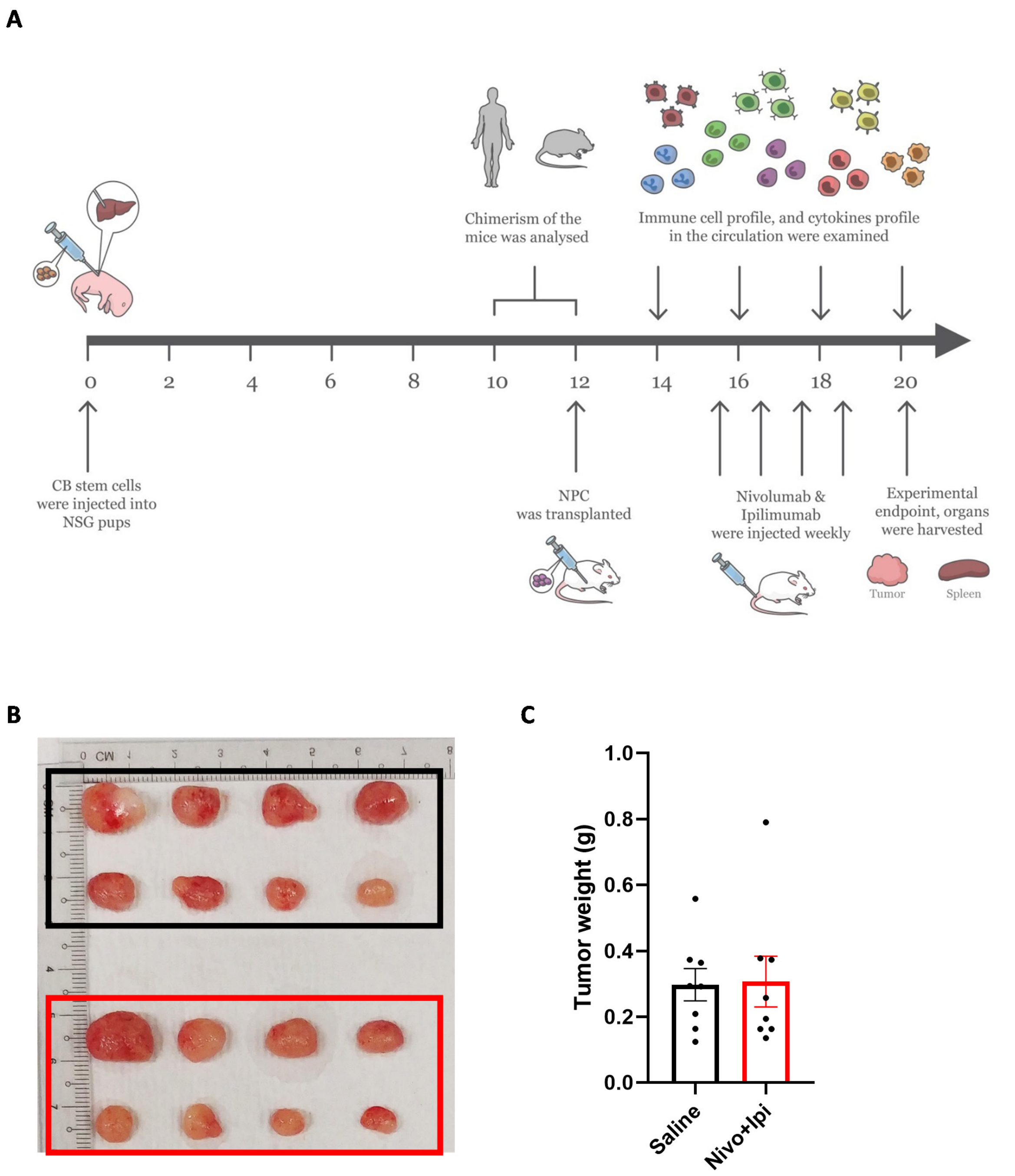

Figure S5. Evaluation of the anti-tumor efficacy of nivolumab and ipilimumab in humanized mice. (**A**) Schematic of tumor engraftment and cancer immunotherapy in humanized mice. (**B**) Representative image of tumors from humanized mice in saline-treated group (*n* = 8; Black box) and ICB-treated group (*n* = 8; Red box). (**C**) The tumor weight from the mice are shown. Data are expressed as means ± SEM.

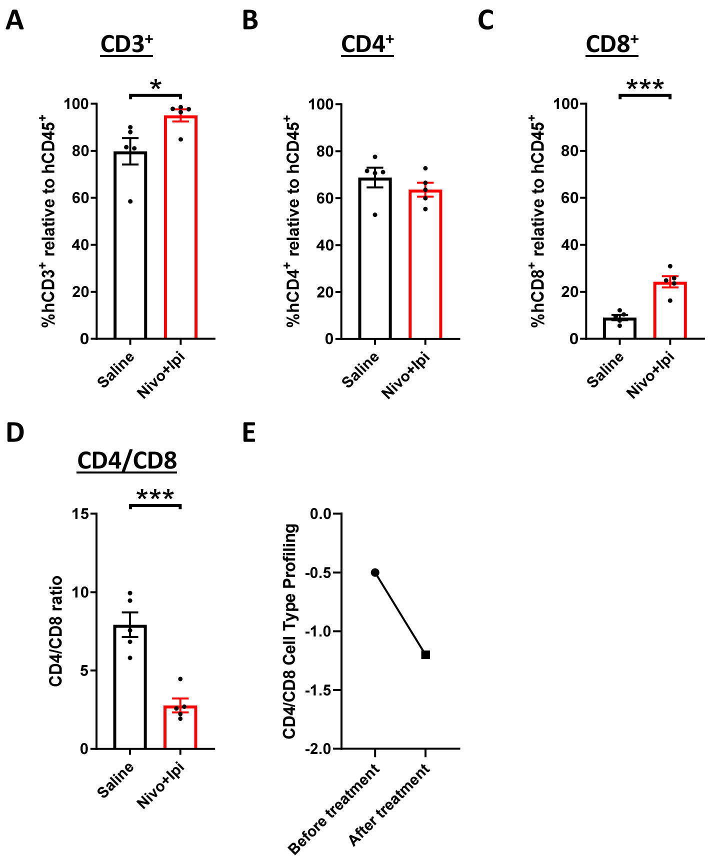

Figure S6. Modulation of immune cells profile in tumor after combination immunotherapy using another NPC-PDX. Tumor were harvested from saline-treated humanized mice ($n = 5$) and nivolumab plus ipilimumab-treated humanized mice ($n = 5$) at the experimental endpoint. The percentage of $CD3^+$ T cells (**A**), $CD4^+$ T cells (**B**), $CD8^+$ T cells (**C**) and CD4/CD8 ratio (**D**) was analyzed. (**E**) The CD4/CD8 cell type profiling from corresponding donor was revealed by Nanostring analysis. Data are expressed as means ± SEM. * $p < 0.05$; *** $p < 0.001$.

Table S1. FACS antibodies used in the present study.

Surface marker antibody	Fluorochrome	Clone	Brand
CD3	BUV737	UCHT1	BD Biosciences
CD4	Bv650	SK3	BD Biosciences
CD8	BUV737	SK1	BD Biosciences
CD14	APC	HCD14	Biolegend
CD16	Bv786	3G8	BD Biosciences
CD19	PE	HIB19	Biolegend
CD45	BUV395	HI30	BD Biosciences
CD45.1	Bv421	A20	BD Biosciences
CD45RA	FITC	HI100	Biolegend
CD56	Bv421	NCAM16.2	BD Biosciences
CD152 (CTLA-4)	PE	BNI3	Biolegend
CD197	Bv510	3D12	BD Biosciences
CD223 (LAG3)	APC	3DS223H	eBioScience
CD279 (PD-1)	Bv711	EH12.1	BD Biosciences
CD366 (TIM3)	Bv786	F38-2E2	Biolegend
HLA-DR	APC-Cy7	L243	BD Biosciences
TIGIT	PE-Cy7	MBSA43	Thermo Fisher Scientific
